# Characterising equine abdominal lipomata: Can histological features improve the understanding of pathogenesis and risk?

**DOI:** 10.1111/evj.14483

**Published:** 2025-02-20

**Authors:** Alexandra Gillen, Debra Archer, Joanne Ireland, Guido Rocchigiani

**Affiliations:** ^1^ Department of Equine Clinical Sciences University of Liverpool Neston UK; ^2^ Department of Vet Anatomy Physiology and Pathology University of Liverpool Neston UK

**Keywords:** colic, histology, horse, lipoma, small intestinal strangulating lesion

## Abstract

**Background:**

Strangulating lipomata are the most common cause of small intestinal strangulating obstruction. Evaluation of histological features of pathological and non‐pathological lipomata, and the histological properties of omental and retroperitoneal fat have not been described.

**Objectives:**

To characterise histological features of equine abdominal lipomata, omental and retroperitoneal adipose tissue, and associations between them.

**Study design:**

Prospective observational anatomic (gross and histological).

**Methods:**

Horses undergoing emergency laparotomy for management of abdominal pain in a single hospital were recruited. Signalment was recorded. Gross features of lipomata that were a cause of strangulating obstruction (pathological lipomata [PAL]), and lipomata that were currently not causing an intestinal obstruction (pedunculated [PEL] or non‐pedunculated [NPL]) were recorded. Lipomata that were removed intra‐operatively, or following owner‐requested euthanasia, as well as omentum or retroperitoneal adipose tissue, where excised routinely as part of routine management (or post‐euthanasia) were fixed in 10% neutral‐buffered formalin prior to staining (haematoxylin and eosin, picrosirius red). Descriptive statistical analyses were performed. Pearson's chi‐square, Fisher's exact or Kruskal–Wallis tests, as appropriate, were used to assess associations. Significance was *p* < 0.05.

**Results:**

Seventy‐four horses were enrolled; 71 lipomata, 48 retroperitoneal adipose samples, and 26 omental samples underwent evaluation. Increasing age was predictive of lipomata presence and PAL/PEL. Neither omental nor retroperitoneal adipose tissue histological features were correlated with lipomata presence or type. PAL were more likely to exhibit capsule formation (PAL: 70%, NPL: 42%, *p* = 0.03), and had a higher vascular density (median 10.6; IQR: 8.8–16.8; *p* = 0.05), compared with NPL. PEL were more likely to exhibit steatonecrosis (PEL: 92%, NPL: 33%, *p* = 0.01) and had increased mineralisation (PEL: 67%, NPL: 17%, *p* = 0.05) compared with NPL.

**Main limitations:**

Small sample size.

**Conclusions:**

Histological features of omental and retroperitoneal fat do not predict presence of lipomata or type. However, there are histological features of PAL and PEL which may be related to pathological potential.

## 1 INTRODUCTION

Abdominal lipomata are common in the horse and are a frequent cause of colic requiring surgical intervention, particularly in the older horse or pony. Accumulations of adipocytes are common within the mesentery or omentum.^1^ Lipomata are benign neoplastic (or hyperplastic) masses that do not infiltrate the surrounding tissues.^2^ However, abdominal equine lipomata often form pedunculated masses.^3^ Lipomata (or a solitary lipoma) can be a cause of intestinal obstruction in a number of ways, most commonly resulting in strangulating obstruction and usually affecting the small intestine. Less commonly the small colon and occasionally other gastrointestinal regions including the rectum, large colon and caecum have been involved.^3^, ^4^, ^5^ This should be differentiated from lipomatosis, which involves extensive adipocytic infiltrate with disruption to the architecture of the affected organ, which has also been reported in the equine gastrointestinal tract but which is less frequent.^6^ In canines and people, abdominal lipomata are rare.^7^, ^8^, ^9^, ^10^, ^11^ Risk factors in canines include breed, neutering and obesity,^12^, ^13^ whereas in people, two‐thirds of lipomata have a direct genetic aetiology, for example, multiple hereditary lipomatosis,^14^ or are related to disorders such as Gardner syndrome or adiposis dolorosa.^15^, ^16^, ^17^, ^18^

Currently, there is limited published information about the histological features of equine abdominal lipomata. Pedunculated and sessile (without a pedicle) lipomata have been described in the equine abdomen but histological features that may distinguish between the two forms, and which plausibly could play a role in lipoma pathogenesis, have not been previously investigated. This is particularly relevant given that pedunculated forms of lipomata are more commonly involved with intestinal strangulation and can be difficult to predict without surgical exploration of the abdomen.^19^ Geldings and increased age are known to increase the risk of colic due to strangulating pedunculated lipomata.^19^, ^20^ However, currently it is not known whether there are other risk factors that can be measured non‐invasively to better predict horses at increased risk of the latter form of colic and potentially be used to reduce the risk of colic where modifiable.

Horses with a higher mesenteric and omental adipose tissue EQUIFAT scores, have been reported to have increased incidence of mesenteric lipoma development.^21^ However, this does not distinguish between the more potentially pathogenic pedunculated forms of lipomata and less pathogenic sessile forms. Although mesenteric and omental adipose tissue can only be sampled surgically, retroperitoneal adipose tissue, the depth of which is measured in the EQUIFAT score, is more superficially located. This tissue could potentially be sampled using minimally invasive percutaneous biopsy techniques, such as those commonly used in horses to obtain samples from organs such as the liver.^22^ Whether or not retroperitoneal adipose tissue can be used to predict the presence of lipomata has not been evaluated. There are currently no studies evaluating the histological properties of retroperitoneal fat and if features of retroperitoneal fat correlate with the pedunculated or non‐pedunculated forms of intra‐abdominal lipomata.

The aim of this study was (1) to characterise in‐depth the histological features of pathological and non‐pathological forms of equine abdominal lipomata and associations with patient signalment, and (2) to determine if histological features of retroperitoneal fat correlate with pathological forms of abdominal lipomata. We hypothesised that histological features would differ between pathological and non‐pathological lipomata, and that the presence of lipomata would vary according to sex, increased age and specific histological features of retroperitoneal fat.

## 2 MATERIALS AND METHODS

This was a prospective study conducted in horses undergoing exploratory laparotomy via ventral midline incision for treatment of colic (acute abdominal pain) at a single equine hospital. Horses were deemed eligible for inclusion if they were presented with colic over a 19‐month period (January 2021–August 2022), were greater than 1 year of age, were deemed to require exploratory laparotomy based on evaluation and consultation with a board‐certified surgeon, and whose owners gave informed consent for enrolment. Determination of the requirement for exploratory laparotomy was determined by the horse's comfort levels, response to analgesia, physical examination including vital parameters, blood and peritoneal fluid parameters, as well as abdominal sonography and transrectal palpation.

Age, breed, sex and the number and type of lipomata identified during surgical assessment were recorded. Lipomata were defined as pathological (PAL: causing a strangulating lesion), pedunculated (PEL: pedunculated, i.e., having a visible fibrous stalk, but not currently causing a strangulating lesion), and non‐pedunculated (NPL: not pedunculated and not currently causing a strangulating lesion). As part of routine surgical management, strangulating lipomata (where present) were excised together with other lipomata that were considered to be a potential cause of future colic episodes, as a preventive measure. In horses euthanased intra‐operatively at the owners' request, lipomata were harvested immediately post‐mortem with owner consent. Lipomata were removed by transecting the pedicle (if present), or transecting the lipoma as close to the mesentery as was deemed appropriate, with Metzenbaum scissors. Ligation with appropriate size absorbable suture material was performed if deemed necessary by the surgeon. Any resulting mesenteric defects were sutured closed using 2–0 poliglecaprone (Ethicon) in a simple interrupted or simple continuous pattern at the discretion of the surgeon. Samples were also taken from any excess retroperitoneal adipose tissue and omentum that were removed according to clinical indication. Retroperitoneal fat was only removed if it was deemed, by the surgeon, to impede closure of the linea alba, or if it was so disrupted during routine handling that a small area risked devitalisation if left in situ. If a small quantity of retroperitoneal fat was removed, this was done manually. Omentectomy, where deemed necessary by the surgeon was performed using emasculators. Once obtained, samples were stored immediately in 10% neutral‐buffered formalin (pH 7.4) solution for at least 24 h, with a 1:10 sample to formalin ratio prior to histological assessment. Samples were trimmed to fit in cassettes, processed routinely to produce 4‐μm thick sections which were stained with haematoxylin and eosin. Picrosirius red stain was used to evaluate the percentage collagen content of the samples.^23^

Histological features assessed are detailed in the scoring system (Table 1). Histological assessment in all samples used a scoring system, with a semi‐quantitative approach (when possible), ranging from 0 (absent) to 3 (largely present), for specific histological features. Binary categorical variables included the presence or absence of the following: a capsule, haematoidin (a golden‐brown pigmentation resulting from haemoglobin metabolism under low oxygen tension),^24^ and mesothelial papillary hyperplasia. Ordinal categorical variables included: haemorrhage, brown pigment laden macrophages (i.e., lipofuscin or/and haemosiderin), steanonecrosis, mineralisation, neutrophilic steatitis, granulomatous steatitis, and thrombosis. Continuous variables evaluated were vascular density (per ×20 high power field (hpf)) and collagen percentage. For the latter parameter, picrosirius red slides were digitalised using scanner Aperio CS2 (Leica Biosystems). Machine learning software (Orbit, Idorsia Pharmaceuticals Ltd.) was used to calculate a percentage collagen of the whole slide.^25^

**TABLE 1:** Scoring system for histological features of lipomata, omental and retroperitoneal adipose tissue.

Categorical factors				
Factor/score	0	1	2	3
Brown pigment laden macrophages	Not present	Scattered macrophages not forming clusters (up to 5 cells)	Multiple macrophages forming variably frequent clusters (5–50 cells)	Numerous macrophages forming sheets of more than 50 cells
Steatonecrosis	0%–5%	5%–25% of the area involved	25%–50% of the area involved	>50% of the area involved
Mineralisation	Not present	Rare and isolated areas of mineralisation	Coalescing areas of mineralisation occupying less than one 10× HPF area	Coalescing areas of mineralisation extending for more than one 10× HPF
Steatitis (neutrophilic or granulomatous)	Not present	Rare and isolated areas of steatitis	Coalescing areas of steatitis occupying less than one 10× HPF area	Coalescing areas of steatitis extending for more than one 10× HPF
Thrombosis	Not present	1–2 vessels showing acute/chronic thrombosis	3–4 vessels showing acute/chronic thrombosis	>4 vessels showing acute/chronic thrombosis
Haemorrhage	Not present	Isolated haemorrhages forming poorly cohesive clusters, mostly perivascular (not extending more than 10×)	Multiple clusters of haemorrhage forming masses extending less than 10× (not restricted around vessels)	Large clusters of haemorrhages extending for more than one 10× HPF
Binary factors				
Capsule	Not present		Present Consisting of at least 5 layers of fibroblasts. If partially capsulated, considered capsulated	
Haematoidin (extracellular golden pigment)	Not present		Present	
Mesothelial papillary hyperplasia	Not present		Present	
Quantitative factors				
Collagen percentage	Assessed in the entire slide, using picrosirius red and calculated as a percentage of the entire tissue examined			
Vascular density	Assessed in five 20× fields as a mean of the five calculations. Two fields evaluated in the capsule and three in the remainder of the lipoma. If a capsule was not present, five fields were evaluated in the remainder of the lipoma			

Statistical analyses were performed using SPSS (IBM Corp). Continuous variables were summarised as means with standard deviation (SD) when Normally distributed, and as medians with interquartile range (IQR) when non‐Normally distributed. Categorical data were presented as percentages with 95% confidence intervals. Pearson's chi‐square or Fisher's exact tests, as appropriate, were used to assess associations between categorical variables. Post hoc *z*‐tests for independent proportions with Bonferroni adjustment for multiple comparisons were used for pairwise comparisons. Kruskal–Wallis tests, with Bonferroni adjustment for post hoc multiple comparisons, were used to compare median values for vascular density and percentage of collagen between lipoma types. A univariable logistic regression model was used to assess associations between signalment and lipoma (binary outcomes of: i, any lipomata; ii, any PAL and/or PEL). Variables with *p* < 0.25 were considered for inclusion in multivariable logistic regression models, built using backwards elimination. Univariable mixed effects logistic regression models were used to identify histological characteristics of retroperitoneal fat or omentum associated with the presence of specific lipoma histological features. Because of the potential for clustering effects (where horses had multiple lipomata), within‐horse clustering was accounted for by incorporation of ‘horse’ as a random intercept term in all models. Correlation between vascular density of retroperitoneal or omental adipose tissue, and lipomata was evaluated with Spearman's rank‐order correlations. Due to a lack of control horses over 20 years of age, age was dichotomised. Previous studies have suggested mean ages of 16.9–19.2 years for horses with strangulating lipomata.^1^, ^19^, ^26^, ^27^ An age slightly younger than these means was therefore selected for dichotomisation. Statistical significance was set at *p* < 0.05.

## 3 RESULTS

### 3.1 Study population

During the study period 74 horses were eligible for study inclusion. The mean age of horses was 15.9 years (SD 5.8). Breed was recorded as ‘unknown’ in seven cases. Breeds included UK and Irish native pony breeds (*n* = 15; 22%), Thoroughbred/Thoroughbred crossbreeds (*n* = 10; 15%), Cob/Cob crossbreeds/Welsh Section D (*n* = 15; 22%), Irish Draught/Irish Draught crossbreeds (*n* = 9; 13%) and other light horse breeds (*n* = 18; 27%). Sex was recorded in 72 cases; of these, 47 (65%) were male and were all geldings apart from one entire male, and 25 (35%) were female.

**FIGURE 1:**
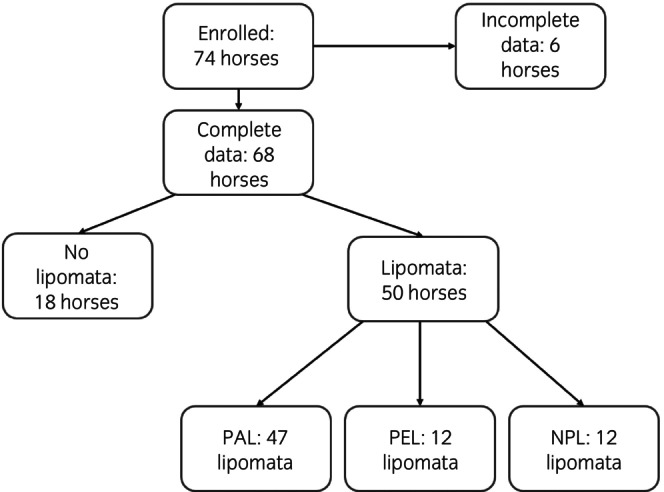
Flow chart showing the number of enrolled horses, and those with and without lipomata, as well as lipoma types. PAL (pathological lipomata, causing a strangulating obstruction at the time of exploratory laparotomy), PEL (pedunculated lipomata, not causing a strangulating obstruction at the time of exploratory laparotomy), NPL (non‐pedunculated lipomata, not causing a strangulating obstruction at the time of exploratory laparotomy).

Complete data for lipomata presence or absence was recorded in 68 horses (lipomata were not removed from all horses due to the proximity of vessels). No lipomata were present in 18 horses (27%), and 50 horses (73%) had one or more abdominal lipoma(ta). Of the cases with at least one lipoma, 46 (92%) had PAL, and or PEL and 4 horses (8%) only had forms that were not currently pathological (not causing a strangulating lesion), and not pedunculated (NPL). Samples were obtained from a total of 71 lipomata from 50 horses classified as PAL (*n* = 47; 66%), PEL (*n* = 12; 17%) and NPL (*n* = 12; 17%). Retroperitoneal adipose tissue was obtained from 48 horses and omentum from 26. Samples of both omentum and retroperitoneal adipose tissue were obtained from 14 horses. A flow chart of horses enrolled in the study is shown in Figure 1.

### 3.2 Histological characteristics of lipomata

**FIGURE 2:**
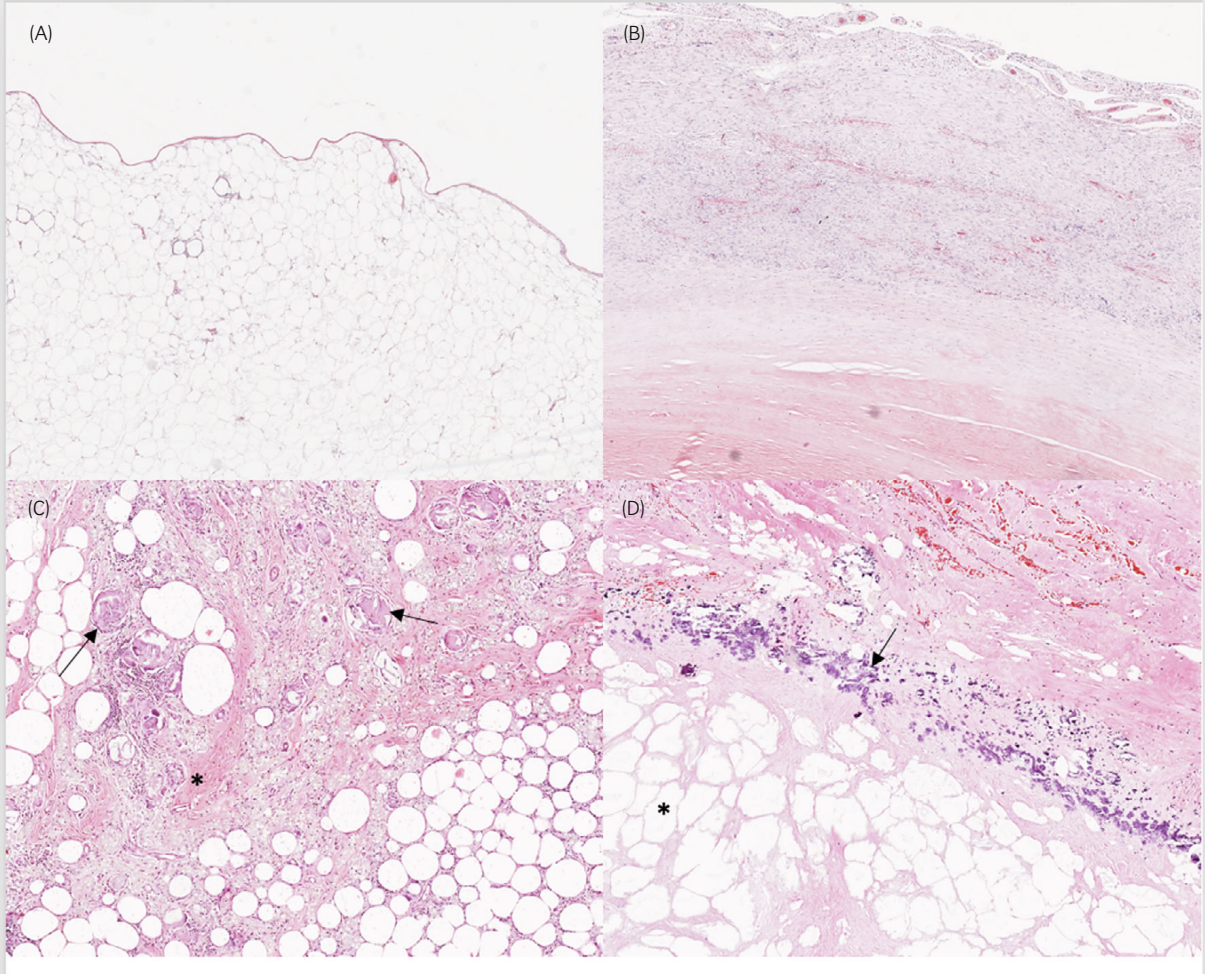
Histological features of abdominal lipomata in horses. Images A and B are at 5× and images C and D at 10× magnification. (A) Prototypical NPL (non‐pedunculated lipoma, not causing a strangulating obstruction at the time of exploratory laparotomy). There is absence of a capsule, while most of the adipocytes within it show minimal atypia or other changes (e.g., fibrosis, necrosis, inflammation) and minimal numbers of vessels. Haematoxylin and eosin (HE). (B) PEL (pedunculated lipoma, not causing a strangulating obstruction at the time of exploratory laparotomy). Severely thickened capsule showing increased vascularity. HE. (C) PEL. Core of the lipoma showing mixed inflammatory changes with numerous macrophages, multinucleated giant cells (arrows) and increased interstitial fibrosis (asterisk). HE. (D) PAL (pathological lipoma, causing a strangulating obstruction at the time of exploratory laparotomy). Core of lipoma showing evident and extensive steatonecrosis (asterisk) and adjacent mineralisation (arrow). HE.

**FIGURE 3:**
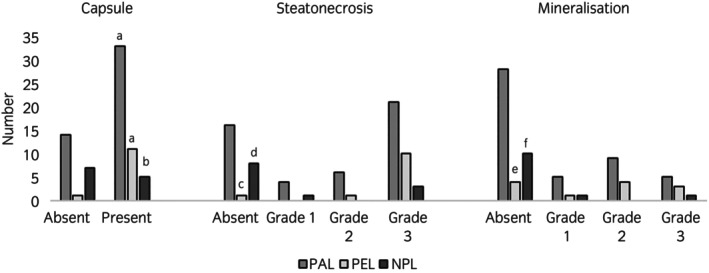
Bar charts showing the presence or absence of a capsule, and the grade of steatonecrosis and mineralisation. PAL (pathological lipoma, causing a strangulating obstruction at the time of exploratory laparotomy) is shown in mid‐grey, PEL (pedunculated lipoma, not causing a strangulating obstruction at the time of exploratory laparotomy) in light‐grey, and NPL (non‐pedunculated lipoma, not causing a strangulating obstruction at the time of exploratory laparotomy) in dark grey. PAL and PEL were significantly more likely to exhibit a capsule compared with NPL (bars denoted ‘a’ are significantly different from bars denoted ‘b’). PEL were significantly more likely to exhibit any grade of steatonecrosis and mineralisation compared with NPL (bars denoted ‘c’ are significantly different from bars denoted ‘d’; bars denoted ‘e’ are significantly different from bars denoted ‘f’).

All histological findings are shown in Tables S1 and S2; significant findings are described below and shown in Figures 2 and 3. A capsule was exhibited in 49 of 71 (69%) lipomata (Figure 2B). Lipoma type was associated with the presence of a capsule (*p* = 0.03), with a lower proportion of NPL exhibiting a capsule compared with PAL or PEL (Figure 3).

**FIGURE 4:**
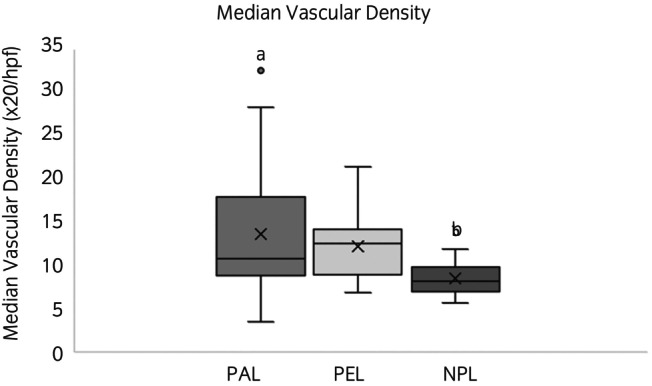
Box and whisker plot showing median vascular density. The boxes represent the interquartile range, the horizontal line the median, and the ‘x’ the mean. PAL (pathological lipoma, causing a strangulating obstruction at the time of exploratory laparotomy) is shown in mid‐grey, PEL (pedunculated lipoma, not causing a strangulating obstruction at the time of exploratory laparotomy) in light‐grey, and NPL (non‐pedunculated lipoma, not causing a strangulating obstruction at the time of exploratory laparotomy) in dark grey. NPL exhibited a significantly lower median vascular density compared with PAL (the bar denoted ‘a’ is significantly different from the bar denoted ‘b’).

Median vascular density (Figure 1B) in lipomata was 10.6 per ×20 high powered field (hpf) (0.78 mm^2^) (IQR 8.4–14.6). Median vascular density was higher in PAL compared with NPL (*p* = 0.05), however, there was no difference between PEL and other lipoma types (Figure 4). Median vascular density did not differ between lipomata with a capsule and those without (*p* = 0.86).

Steatonecrosis was observed in 64.8% (*n* = 46/71) of lipomata (Figure 2C,D). Lipoma type was associated with the presence of steatonecrosis (*p* = 0.01), with steatonecrosis being observed in a higher proportion of PEL compared with PAL or NPL (Figure 3).

Overall, 40.8% (*n* = 29/71) of lipomata had evidence of mineralisation. Lipoma type was associated with the presence of mineralisation, with a higher proportion of PEL exhibiting evidence of mineralisation (*p* = 0.05) compared with PAL or NPL (Figure 3).

### 3.3 Associations between histological characteristics of retroperitoneal adipose tissue and lipomata

Of the 48 horses from which a retroperitoneal adipose sample was obtained, 25 (52%) had samples from at least one lipoma. There were no significant associations between the histological characteristics of retroperitoneal adipose tissue and the presence of lipomata of any type, or the presence of PAL and PEL (all features *p* > 0.05). This remained true when assessing clustering at horse level in mixed effects logistic regression. Haemorrhage in retroperitoneal adipose tissue was positively associated with haemorrhage in lipomata (odds ratio [OR] 15.8; 95% CI 1.7–146.7; *p* = 0.02). No other retroperitoneal adipose histological characteristics were significantly associated with the presence of any lipoma histological characteristics.

### 3.4 Associations between histological characteristics of omental adipose tissue and lipomata

Of the 26 horses from which an omental adipose sample was obtained, 15 (58%) had samples from at least one lipoma. There were no significant associations between the histological characteristics of omental adipose tissue and the presence of lipoma of any type, or the presence of PAL and PEL. There were also no significant associations between omental histological characteristics and the presence of any lipoma histological characteristics.

### 3.5 Risk factors for lipomata

Results of univariable logistic regression analyses for the presence of lipoma(ta) of any type and the presence of PAL or PEL are displayed in Table 2. Age ≥15 years was significantly associated with increased odds of any lipomata (OR 9.62; 95% CI 2.77–33.43; *p* < 0.001) or odds of a PAL or PEL compared with no lipomata or only NPL (OR 7.78; 95% CI 2.42–24.96; *p* < 0.001; Table 2). Ponies were more likely to have lipomata compared with Thoroughbreds and Thoroughbred crosses (OR 21.00; 95% CI 1.92–229.39; *p* = 0.04). Ponies, Draught breeds, and horses that were not Thoroughbreds or Draught breeds had increased odds of exhibiting PAL or PEL compared with no lipomata or only NPL (OR 32.67; 95% CI 2.85–374.14; *p* = 0.005; OR 16.33; 95% CI 1.35–197.77; *p* = 0.028; OR 6.70; 95% CI 1.12–33.24; *p* = 0.04, respectively). Sex was not associated with the presence or type of lipomata. Mixed effects logistic regression revealed no evidence of clustering. When performing multivariable analyses, with both presence of any lipomata, and presence of PAL/PEL as the dependent variable, only age remained in the model.

**TABLE 2:** Univariable logistic regression of signalment and factors associated with presence of any lipoma type (Model 1) and presence of strangulating lesion (PAL) or non‐strangulating pedunculated (PEL) lipomata (Model 2).

Model 1						
Variable (*n*)	Category (*n*)	No lipoma % (*n*)	Lipoma of any type % (*n*)	Odds ratio	95% CI for odds ratio	*p* value
Age	<15 years	56.5 (13)	43.5 (10)	Reference		<0.001
	≥15 years	11.9 (5)	88.1 (37)	9.62	2.77–33.43	
Sex	Male	27.3 (12)	72.7 (32)	Reference		0.9
	Female	28.6 (6)	71.4 (15)	0.94	0.29–2.98	
Breed	Thoroughbred/Thoroughbred crosses	60.0 (6)	40.0 (4)	Reference		0.04
	Other light horse >14.2hh	27.8 (5)	72.2 (13)	3.90	0.76–19.95	
	Irish Draught/Irish Draught crosses	12.5 (1)	87.5 (7)	10.50	0.91–121.39	
	Cob/Cob crosses/Welsh Section D	35.7 (5)	64.3 (9)	2.70	0.51–14.37	
	Pony	27.2 (1)	72.3 (14)	21.00	1.92–229.39	

Model 2						
Variable (*n*)	Category (*n*)	No lipoma or only NPedL	PathL or PedL	Odds ratio	95% CI for odds ratio	*p* value
Age	<15 years	60.9 (14)	39.1 (9)	Reference		<0.001
	≥15 years	16.7 (7)	83.3 (35)	7.78	2.42–24.96	
Sex	Male	31.8 (14)	68.2 (30)	Reference		0.9
	Female	33.3 (7)	66.7 (14)	0.93	0.31–2.82	
Breed	Thoroughbred/Thoroughbred crosses	70.0 (7)	30.0 (3)	Reference		0.02
	Other light horse >14.2hh	27.8 (5)	72.2 (13)	6.70	1.12–33.24	
	Irish Draught/Irish Draught crosses	12.5 (1)	87.5 (7)	16.33	1.35–197.77	
	Cob/Cob crosses/Welsh Section D	50.0 (7)	50.0 (7)	2.33	0.42–12.91	
	Pony	27.2 (1)	72.3 (14)	32.67	2.85–374.14	

## 4 DISCUSSION

This study is the first comprehensive description of the histological characteristics of pathological (PAL) and not currently pathological (PEL and NPL) forms of equine abdominal lipomata, equine retroperitoneal adipose tissue, and equine omentum and has demonstrated the key features of PAL, PEL and NPL forms. The results of this study may provide avenues for further research, leading to a better understanding of the mechanisms of lipomata formation.

Differences between pedunculated (PEL) and non‐pedunculated (NPL) lipomata include steatonecrosis, mineralisation, and the presence of a capsule. These findings may offer hypotheses as to the formation or the consequence of pedicle formation. Steatonecrosis and mineralisation can occur due to a lack of blood supply to adipose cells.^28^ This is rarely reported in benign subcutaneous masses in other species but it is reported in large abdominal and thoracic lipomata in canines.^9^, ^10^ The human literature on infiltrative lipomata and other lipomata causing significant pathology report rare instances of steatonecrosis and mineralisation.^29^, ^30^, ^31^, ^32^ However, it must be appreciated that many of these examples, due to species and mass location, may lack applicability to equine abdominal lipomata. It is possible that the presence of steatonecrosis and mineralisation increase the weight of a lipoma, potentially making the lipoma more likely to form a pedicle. This is supported by a previous retrospective study where lipomata currently causing a strangulating obstruction had a higher weight compared with those not currently causing a strangulating obstruction.^19^ Conversely, an alternative hypothesis is that the presence of a pedicle may result in reduced bloodflow to the adipocytes, thereby predisposing to steatonecrosis. Interestingly, this study found an almost complete lack of thrombosis in the majority of lipomata. This finding seems to exclude a prominent role of thrombosis in the pathogenesis of steatonecrosis or any other degenerative process occurring within abdominal lipomata in horses. The authors therefore hypothesise that once steatonecrosis occurs, secondary calcification can commence, resulting in mineralisation.^33^ It is therefore likely that mineralisation occurs secondary to steatonecrosis, potentially explaining why both features are more common in PEL compared with NPL.

The present study found PAL and PEL were more likely to exhibit a capsule compared with NPL. Although abdominal lipomata in human patients are rare, they typically exhibit a fibrous capsule.^15^, ^34^, ^35^ Moreover, the majority of pedunculated lipomata in people, regardless of location, exhibit a capsule.^36^, ^37^ Although uncommonly reported, abdominal lipomata in canines can exhibit a capsule, as well as containing necrotic tissue.^9^, ^10^ However, it is not possible, based on findings from this study, to determine whether or not a capsule makes pedicle formation more likely. This would, however, be an area for further investigation.^3^

Acute haemorrhage, brown‐pigment laden macrophages (mainly haemosiderophages), steatonecrosis, inflammation, mineralisation and haematoidin were present in multiple lipomata examined in the present study. The presence of haemoglobin breakdown products (i.e., haematoidin, haemosiderin) within the lipomata indicates that, at some point during development, haemorrhage occurred. This may suggest that chronic haemorrhage (and steatonecrosis), inflammation and fibrosis are consequential steps of the ‘maturation’ of some lipomata, as reported in human fat necrosis.^38^ Lipomata showing haematoidin and mineralisation should therefore be considered chronic in nature. In the present study, PAL interestingly showed minimal evidence of such chronic features, therefore, suggesting that although long‐standing lipomata may be more likely to form a pedicle, they are not always those responsible for small intestinal strangulating lesions.

Pathological lipomata (PAL) demonstrated an increased median vascular density compared with NPL. Literature discussing the vascular density of lipomata is minimal. In one human study, vascular density in lipomata was not different to that of adipose tissue.^39^ However, studies comparing lipoma and liposarcoma, or atypical lipomatous tumours, found increased vascularity in liposarcoma and atypical lipomatous tumours, compared with lipomata.^40^, ^41^ At this stage, little is known about the vascularity of equine abdominal lipomata, and pathogenicity is likely multifactorial, however, it is possible that a higher vascular density may contribute to pathogenicity. Another possible explanation is that the increased vascularisation is secondary to the steatonecrosis and other pathological processes occurring in pathological lipomata, being more an effect rather than a cause of the more active behaviour of these masses.^42^

Histological features of omentum and retroperitoneal adipose tissue were not predictors of the presence of lipomata, or of the type of lipomata found. Haemorrhage was observed in both retroperitoneal adipose tissue and lipomata samples, however, it is likely that this occurred at the time of sampling of retroperitoneal fat. This is interpreted as evidence of similar vascular fragility in retroperitoneal adipose tissue and in lipomata. This is unsurprising, given that both tissues are composed primarily of adipose tissue.^2^ It is therefore the authors' opinion that haemorrhage in retroperitoneal tissue cannot be considered predictive of the presence of lipomata.

This study suggests that there is no relationship between the histological features of omental or retroperitoneal adipose tissue and the presence of lipomata. Previous evidence suggests lipomata formation is complex and likely to have an endocrine component.^43^ Therefore, other avenues, such as lipidomics, which can assess metabolic pathways in lipids should be pursued.

Consistent with previous research, horses aged 15 years or over had increased odds of lipomata formation compared with horses less than 15 years of age.^19^ Of note, horses aged 15 years or over also had increased odds of having lipomata that were currently causing a strangulating lesion or that were pedunculated.^3^ Pathological lipomata (PAL) occurring mostly in older horses has been well‐documented, while the higher prevalence of PAL and PEL in horses over 15 years of age has not been reported previously.^19^, ^20^

In the current study, ponies were at increased risk of development of lipomata compared with Thoroughbred and Thoroughbred crosses. A previous retrospective study also found PAL were more common in ponies compared with Thoroughbred and Thoroughbred crosses.^19^ Interestingly, Draught breeds, ponies and other breeds of horse that were not Thoroughbred or Thoroughbred crosses, were at increased odds of developing PAL and PEL. Given the differing histological features of PAL and PEL compared with NPL, and the fact that people can exhibit a genetic predisposition for development to lipomata,^15^, ^18^ further investigation into the effect of breed should be undertaken.

Limitations of this study include the small sample size, particularly the small number of NPL and PEL. Given the small size of some NPL and the frequency with which they occur in some horses, it was not appropriate to remove all NPL observed due to an inability to safely remove all lipomata and the additional surgical time this would incur. Only horses requiring surgical intervention for colic were evaluated, resulting in inherent bias. A lack of aged control horses without lipomata limits the ability to evaluate age as a variable with which to predict the presence of lipomata. Since this was a small analytical study using a convenience sample, where all eligible cases were included, a sample size calculation was not performed. As this is an initial description of equine abdominal lipomata, the grading system was based on documenting all features observed in lipomata; this resulted in the need to use a novel scoring system. Systemic disease and metabolic status of the horse were not detailed in this study. Lacking this knowledge can make histological characteristics of retroperitoneal adipose tissue, such as necrosis and mineralisation, challenging to interpret. This study did not evaluate the metabolic features of lipomata; lipidomics is an ongoing area of study. Finally, PAL included lipomata that may not have been pedunculated. Due to the nature of strangulating lesions and the techniques sometimes employed to reduce a strangulating lesion, it was often not possible to confirm whether or not a lipoma which was causing a strangulating lesion had a pedicle.

In conclusion, equine abdominal lipomata exhibit many of the features of pathological lipomata that have been observed in people and canines. The majority of PAL have a higher median vascular density, whereas PEL were more likely to exhibit mineralisation and steatonecrosis compared with NPL. The majority of equine abdominal lipomata exhibited features of chronicity but this did not correlate with those lipomata already causing a strangulating lesion. However, as formation of a pedicle is associated which chronicity, surgeons should consider removal of lipomata where it is safe to do so. Breed and more advanced age are predictive of PAL and PEL forms of abdominal lipomata. Further evaluation of metabolic features of fat and association with increasing age, breed type and sex are warranted. Additional investigations, particularly in the field of lipidomics may develop our understanding of lipomata further.

## FUNDING INFORMATION

This study was supported by the Arden and Claudia Sims Lipoma Foundation.

## CONFLICT OF INTEREST STATEMENT

The authors declare no conflicts of interest.

## AUTHOR CONTRIBUTIONS

**Debra Archer:** Conceptualization; funding acquisition; writing – review and editing; methodology; supervision. **Joanne Ireland:** Formal analysis; writing – review and editing. **Guido Rocchigiani:** Conceptualization; investigation; methodology; writing – review and editing. **Alexandra Gillen:** Conceptualization; investigation; writing – original draft; methodology; funding acquisition.

## DATA INTEGRITY STATEMENT

Alex Gillen had full access to all the data in the study and takes responsibility for the integrity of the data and the accuracy of data analysis.

## ETHICAL ANIMAL RESEARCH

The study protocol was approved by the University of Liverpool Ethics Committee: VREC1309.

## INFORMED CONSENT

Consent was obtained via the Leahurst Equine Hospital research consent form.

## Supporting information


**Table S1:** Histological assessments made, and the presence or absence, and grade of those features in different lipoma types. For comparisons using nominal data, only *p* values evaluating the presence or absence of a feature are shown. For continuous variables, median and interquartile ranges are stated.

**Table S2:** Histological assessments made, and the presence or absence, and grade of those features in retroperitoneal and omental adipose tissue. For comparisons using nominal data, only *p* values evaluating the presence or absence of a feature are shown. For continuous variables, median and interquartile ranges are stated.

## Data Availability

The data that support the findings of this study are available from the corresponding author upon reasonable request: Open sharing exemption granted by the editor due to lack of provision in the owner informed consent process.
