# Supplementary material for: Characterising equine abdominal lipomata: Can histological features improve the understanding of pathogenesis and risk?
Source: Equine Vet J. 2025 Feb 20;57(6):1490–9. doi: 10.1111/evj.14483 (PMC12508287; doi:10.1111/evj.14483)
Supplement: Supplementary file 1 — Table S1: Histological assessments made, and the presence or absence, and grade of those features in different lipoma types. For comparisons using nominal data, only p values evaluating the presence or absence of a feature are shown. For continuous variables, median and interquartile ranges are stated. [file EVJ-57-1490-s001.pdf]

Equine Veterinary Journal BEVA

Table S1: Histological assessments made, and the presence or absence, and grade of those features in different lipoma types. For comparisons using nominal data, only *P* values evaluating the presence or absence of a feature are shown. For continuous variables, median and interquartile ranges are stated.

Variable (n)	Category (n)	Frequency (%)			P value
		PAL% (n)	PEL% (n)	NPL% (n)	
Capsule (71)	Absent (22)<br>Present (49)	29.8 (14)<br>70.2 (33)	0.08 (1)<br>91.6 (11)	58.3 (7)<br>41.5 (5)	0.03
Haemorrhage (71)	Absent (26)<br>Present (45)<br>Grade 1 (22)<br>Grade 2 (13)<br>Grade 3 (10)	36.2 (17)<br>63.8 (30)<br>31.9 (15)<br>25.0 (8)<br>33.3 (7)	25.0 (3)<br>75.0 (9)<br>17.0 (3)<br>33.3 (4)<br>8.3 (2)	50.0 (6)<br>50.0 (6)<br>14.9 (4)<br>16.7 (1)<br>8.3 (1)	0.5
Brown pigment macrophages (71)	Absent (22)<br>Present (49)<br>Grade 1 (24)<br>Grade 2 (14)<br>Grade 3 (11)	36.2 (17)<br>63.8 (30)<br>31.9 (15)<br>14.9 (7)<br>17.0 (8)	8.3 (1)<br>91.7 (11)<br>8.3 (3)<br>25.0 (6)<br>50.0 (2)	33.3 (4)<br>66.7 (8)<br>50.0 (6)<br>8.3 (1)<br>8.3 (1)	0.2
Steatonecrosis (71)	Absent (25)<br>Present (46)<br>Grade 1 (5)<br>Grade 2 (7)<br>Grade 3 (34)	34.0 (16)<br>66.0 (31)<br>8.5 (4)<br>12.8 (6)<br>44.7 (21)	8.3 (1)<br>91.7 (11)<br>0 (0)<br>8.3 (1)<br>83.0 (10)	66.7 (8)<br>33.3 (4)<br>8.3 (1)<br>0 (0)<br>25.0 (3)	0.01
Mineralisation (71)	Absent (42)<br>Present (29)<br>Grade 1 (7)<br>Grade 2 (13)<br>Grade 3 (9)	59.6 (28)<br>40.4 (19)<br>10.6 (5)<br>19.1 (9)<br>10.6 (5)	33.3 (4)<br>66.7 (8)<br>8.3 (1)<br>33.3 (4)<br>25.0 (3)	83.3 (10)<br>16.7 (2)<br>8.3 (1)<br>0 (0)<br>8.3 (1)	0.05

Neutrophilic Steatitis (71)	Absent (66)<br>Present (5)<br>Grade 1 (3)<br>Grade 2 (2)<br>Grade 3 (0)	93.6 (44)<br>8.5 (4)<br>23.5 (2)<br>23.5 (2)	100 (12)<br>0 (0)<br>0 (0)<br>0 (0)	91.7 (11)<br>8.3 (1)<br>8.3 (1)<br>0 (0)	0.8
Granulomatous Steatitis (71)	Absent (24)<br>Present (47)<br>Grade 1 (17)<br>Grade 2 (20)<br>Grade 3 (10)	59.6 (15)<br>40.4 (32)<br>23.4 (11)<br>27.7 (13)<br>17.0 (8)	33.3 (4)<br>66.7 (8)<br>8.3 (1)<br>41.7 (5)<br>16.7 (2)	83.3 (5)<br>16.7 (7)<br>41.7 (5)<br>16.7 (2)<br>0 (0)	0.8
Thrombosis (71)	Absent (64)<br>Present (7)<br>Grade 1 (2)<br>Grade 2 (3)<br>Grade 3 (2)	89.3 (42)<br>10.6 (5)<br>4.3 (2)<br>4.3 (2)<br>2.1 (1)	91.7 (11)<br>8.3 (1)<br>0 (0)<br>8.3 (1)<br>0 (0)	91.7 (11)<br>8.3 (1)<br>0 (0)<br>0 (0)<br>8.3 (1)	0.1
Mesothelial papillary hyperplasia (70)	Absent (54)<br>Present (16)	80.4 (37)<br>21.8 (10)	66.7 (8)<br>33.3 (4)	75.0 (9)<br>16.7 (2)	0.7
Haematoidin (71)	Absent (62)<br>Present (9)	87.2 (41)<br>12.8 (6)	83.3 (10)<br>16.7 (2)	91.7 (11)<br>8.3 (1)	0.9
**Variable (n)**		**Median and Interquartile Range**			
		**PedL**	**PedL**	**PedL**	
Median Collagen% (71)		11.1; 2.0 – 28.0	24.7; 17.9 – 33.1	3.9; 2.1 – 22.7	0.2
Median Vascular Density (71)		10.6; 8.8 – 16.8	12.4; 8.8 – 14.0	8.2; 6.9 – 10.4	0.05<br>0.2
