# Supplementary material for: Characterising equine abdominal lipomata: Can histological features improve the understanding of pathogenesis and risk?
Source: Equine Vet J. 2025 Feb 20;57(6):1490–9. doi: 10.1111/evj.14483 (PMC12508287; doi:10.1111/evj.14483)
Supplement: Supplementary file 2 — Table S2: Histological assessments made, and the presence or absence, and grade of those features in retroperitoneal and omental adipose tissue. For comparisons using nominal data, only p values evaluating the presence or absence of a feature are shown. For continuous variables, median and interquartile ranges are stated. [file EVJ-57-1490-s002.pdf]

Table S2: Histological assessments made, and the presence or absence, and grade of those features in retroperitoneal and omental adipose tissue. For comparisons using nominal data, only *P* values evaluating the presence or absence of a feature are shown. For continuous variables, median and interquartile ranges are stated.

**Retroperitoneal adipose tissue variable (n)**	**Retroperitoneal adipose tissue Category %(n)**	**Association with lipoma presence *P* value**	**Omental adipose tissue variable (n)**	**Omental adipose tissue Category %(n)**	**Association with lipoma presence *P* value**
Haemorrhage (48)	Absent (12)<br>Present (36)<br>Grade 1 (23)<br>Grade 2 (9)<br>Grade 3 (4)	0.6	Haemorrhage (26)	Absent (8)<br>Present (18)<br>Grade 1 (15)<br>Grade 2 (2)<br>Grade 3 (1)	>0.9
Brown pigment macrophages (48)	Absent (39)<br>Present (9)<br>Grade 1 (8)<br>Grade 2 (1)<br>Grade 3 (0)	>0.9	Brown pigment macrophages (26)	Absent (16)<br>Present (10)<br>Grade 1 (8)<br>Grade 2 (1)<br>Grade 3 (1)	0.6
Steatonecrosis (48)	Absent (47)<br>Present (1)<br>Grade 1 (1)<br>Grade 2 (0)<br>Grade 3 (0)	>0.9	Steatonecrosis (26)	Absent (22)<br>Present (4)<br>Grade 1 (2)<br>Grade 2 (2)<br>Grade 3 (0)	>0.9
Mineralisation (48)	Absent (47)<br>Present (1)<br>Grade 1 (1)<br>Grade 2 (0)<br>Grade 3 (0)	>0.9	Mineralisation (26)	Absent (26)<br>Present (0)	NA

<table>
<tr><td>Neutrophilic Steatitis (48)</td><td colspan="2">Absent (46)
Present (2)
Grade 1 (1)
Grade 2 (1)
Grade 3 (0)</td><td>>0.9</td><td>Neutrophilic Steatitis (26)</td><td colspan="2">Absent (24)
Present (2)
Grade 1 (1)
Grade 2 (1)
Grade 3 (0)</td><td>v</td></tr>
<tr><td>Granulomatous Steatitis (48)</td><td colspan="2">Absent (37)
Present (11)
Grade 1 (9)
Grade 2 (2)
Grade 3 (0)</td><td>0.4</td><td>Granulomatous Steatitis (26)</td><td colspan="2">Absent (11)
Present (15)
Grade 1 (10)
Grade 2 (3)
Grade 3 (2)</td><td>0.7</td></tr>
<tr><td>Thrombosis (48)</td><td colspan="2">Absent (48)
Present (0)</td><td>NA</td><td>Thrombosis (26)</td><td colspan="2">Absent (17)
Present (1)
Grade 1 (1)
Grade 2 (0)
Grade 3 (0)</td><td>0.5</td></tr>
<tr><td>Mesothelial papillary hyperplasia (48)</td><td colspan="2">Absent (37)
Present (11)</td><td>0.7</td><td>Mesothelial papillary hyperplasia (26)</td><td colspan="2">Absent (0)
Present (26)</td><td>>0.9</td></tr>
<tr><td>Haematoidin (48)</td><td colspan="2">Absent (48)
Present (0)</td><td>NA</td><td>Haematoidin (26)</td><td colspan="2">Absent (26)
Present (0)</td><td>NA</td></tr>
<tr><td>Variable (n)</td><td></td><td>IQR</td><td></td><td>Variable (n)</td><td></td><td>IQR</td><td></td></tr>
<tr><td>Median Vascular Density (48)</td><td>11.4</td><td>7.9-13.8</td><td>0.9</td><td>Median Vascular Density (26)</td><td>26.1</td><td>15.7-36.4</td><td>0.9</td></tr>
</table>
